# Comprehensive metabolomic and microbial analysis of tobacco rhizosphere soil responses to crop rotation and fertilization

**DOI:** 10.3389/fpls.2025.1595870

**Published:** 2025-06-03

**Authors:** Xinyu Liu, Ying Qiao, Xianzhi Wu, Xuanxuan Chen, Fan Yang, Hao Li, Chuanzong Li, Yong Yang, Chunlei Yang, Jun Yu, Pan Luo

**Affiliations:** ^1^ State Key Laboratory of Biocatalysis and Enzyme Engineering, College of Life Sciences, Hubei University, Wuhan, China; ^2^ Bijie Academy of Agricultural Sciences, Bijie, China; ^3^ Tobacco Research Institute of Hubei Province, Wuhan, China

**Keywords:** tobacco, crop rotation, rhizosphere metabolites, microbial diversity, soil fertility

## Abstract

Tobacco (*Nicotiana tabacum* L.) is a crucial Solanaceae crop globally, but its continuous cultivation can lead to soil degradation. Crop rotation offers numerous benefits, including enhanced soil fertility, improved microbial communities, and pest control. However, how different tobacco planting systems specifically reshape rhizosphere metabolite profiles and regulate microbial diversity remains unclear. Here, we analyzed soil samples from four tobacco cropping systems using non-targeted metabolomics, 16S rRNA and ITS sequencing. The results revealed distinct changes in soil metabolite profiles and microbial communities under different treatments. We identified significant alterations in lipid metabolism, amino acid biosynthesis, and secondary metabolite pathways, which influence soil microbial populations and tobacco plant health. Lipid metabolites, including fatty acids and eicosanoids, were particularly notable for their roles in microbial signaling and plant defense. Furthermore, microbial gene abundance analysis indicated that different treatments fostered unique microbial populations, including increased arbuscular mycorrhizal fungi and saprotrophic fungi, which support nutrient cycling and plant growth. These findings highlight the critical interplay between soil metabolites, microbial diversity, and plant productivity, offering insights into optimizing tobacco cropping systems for improved soil health and sustainable agricultural practices.

## 1 Introduction

Tobacco (*Nicotiana tabacum* L.), a member of the Solanaceae family, is one of the most important crops worldwide due to its economic value (Chen et al., 2022). While continuous tobacco cropping offers advantages such as labor savings, reduced working time, and lower production costs, it can also cause long-term soil degradation (Xiong et al., 2023). To mitigate these issues, crop rotation is a common practice that alters the dynamics of tobacco rhizosphere metabolites, impacting soil fertility (Zhou et al., 2023a). Rotation improves soil organic matter content by promoting the decomposition of previous crop residues and enhancing the soil microbial community (Tiemann et al., 2015). It also optimizes soil physical and chemical properties, such as pH and structure, and increases nutrient availability, improving overall nutrient cycling (Behnke et al., 2021). Furthermore, crop rotation helps control soil-borne diseases and pests, ensuring a healthier growth environment for tobacco plants (Lu et al., 2022).

Strip intercropping, a synergistic agroecological system, stands as one of the most efficacious strategies for sustainable yield intensification. *Vulpia myuros* (L.) C.C. Gmelin is a commonly utilized plant for intercropping with crops due to its ecological benefits. Intercropping tobacco with *V. myuros* offers several benefits, including improved soil structure, suppression of weed growth, better regulation of soil nutrients, and reduced pest and disease incidence (Zhang et al., 2020). Similarly, rotating tobacco with crops like rape (*Brassica napus*) enhances soil health, provides pest control, and boosts economic returns (Liu et al., 2024a). However, excessive use of compound fertilizers in tobacco farming can lead to nutrient accumulation, particularly nitrogen and phosphorus, causing soil pollution over time (Ahmed et al., 2017).

The rotation of tobacco with *V. myuros* and rape has distinct effects on soil health, pest and disease control, and economic benefits (Fang et al., 2016; Zhang et al., 2020). These differences reflect variations in soil fertility, pest management strategies, cost-efficiency, and ecological sustainability (Liu et al., 2024b). In tobacco cultivation, two commonly used types of fertilizers are compound fertilizers and fermented cake fertilizers (Jiang et al., 2022). Compound fertilizer refers to a chemical fertilizer containing at least two of the three primary nutrients-nitrogen (N), phosphorus (P), and potassium (K) (Chen et al., 2024a). It is manufactured through chemical synthesis or physical blending, characterized by high nutrient density, minimal secondary components, and stable physicochemical properties. Compound fertilizers, with their balanced nutrient composition, provide rapid and stable fertilization, but their long-term use can degrade soil structure and, if misapplied, may cause seedling damage (Jiang et al., 2022). Fermented cake fertilizer refers to an organic fertilizer produced through microbial fermentation and decomposition of oilseed residues after oil extraction (such as Brassica napusseed cake, soybean cake, and peanut cake). This material serves dual functions by providing organic matter and essential nutrients (e.g., nitrogen, phosphorus, and potassium), thereby enhancing soil fertility and plant growth. Fermented cake fertilizers, on the other hand, improve soil fertility and structure and promote microbial activity. Though their effects are slower, they enhance the aroma and quality of tobacco (Feng et al., 2024). However, their use requires careful management due to their lower nutrient content and cumbersome application process (Feng et al., 2024).

Soil rhizosphere metabolites and microbial communities play a crucial role in linking soil health and crop productivity (Bhattacharyya and Jha, 2012). The impact of different planting methods on tobacco growth is largely mediated by changes in soil metabolites and microbial populations (Sun et al., 2023a). Understanding the relationship between soil metabolites, microbial diversity, and the functional capabilities of microbial communities could offer valuable insights into the advantages of various planting systems for tobacco cultivation (Holden, 2019).

This study investigated four tobacco cropping systems through integrated rhizosphere analysis. Soil samples were systematically collected from distinct cultivation regimes, employing non-targeted metabolomics to characterize metabolites alongside 16S (16S rRNA) and ITS (Internal Transcribed Spacer) amplicon sequencing for deciphering microbial diversity and identifying keystone taxa. Fertilization and intercropping systems may differentially regulate soil microbiome functions. Fermented cake fertilizer enhances bacterial/fungal richness through high active carbon input. V.myuros intercropping optimizes nitrogen cycling and carbon transformation through plant microbe interactions, while rapeseed rotation promotes phosphorus activation and pathogen inhibition. Microbial function differences are driven by lignin/cellulose ratio. The rationale for comparing key metabolites and microbial interactions lies in their dual role as indicators and drivers of soil health. By elucidating how agricultural practices remodel rhizosphere metabolic landscapes and microbial consortia, this study systematically deciphers the mechanisms underlying soil-microbe-plant interactions, providing a new perspective to optimize tobacco cropping systems for enhanced soil functionality and ecological sustainability.

## 2 Materials and methods

### 2.1 Plant materials, growing conditions and treatments

The location of the test is in Xijiadian County (111.18354°E, 32.748336°N), Danjiangkou City, Hubei Province, China. The soil type is classified as yellow-brown earth. Tobacco cultivar of the Yunyan 87 (*Nicotiana tabacum* cv. Yunyan 87) was provided by Wuhan Tobacco Research Institute, Hubei Province. Rhizosphere soil samples were systematically collected using a standardized five-point sampling protocol. For each experimental group, a minimum of six individual plants were selected to ensure representative sampling. Following collection, all samples were promptly transferred to the laboratory under cryogenic conditions and preserved at −80°C to maintain biochemical integrity prior to downstream analyses.

Control treatment (CK): In the CK, ridge cultivation is performed with conventional fertilization to meet the nutrient requirements of tobacco cultivation. The conventional fertilization treatment was a compound fertilizer with nitrogen of 16 kg (35 kg of urea, 46% of nitrogen), P_2_O_5_ of 5.4 kg (30 kg calcium superphosphate, 18% of phosphorus) and K_2_O of 5 kg (10 kg potassium sulfate, 50% of K_2_O) and applied as 75 kg ha^–1^ during the growing season. Additionally, the compound fertilizer was applied at a rate of 1.5 kg per row, with rows spaced 1.2 m apart. Fertilizers were uniformly broadcasted and incorporated into the top 20 cm soil layer during ridge preparation, consistent with regional agricultural practices. Plot - based positioning is adopted, and monitoring is continuously carried out over a five - year period (Zhu et al., 2023).

Treatment 1 (T1, rotation + intercropping group): During late September 2021, *V. myuros* is sown through broadcast seeding at a rate of 300 g of seeds per plot. Subsequently, in late March 2022, the land is plowed and ridges are formed during the fertilization process, with 1.0 kg of compound fertilizer per row. In June 2022, the desiccated above - ground components of *V. myuros* among the ridges are relocated onto the ridges through hoeing for soil - heaping. Each year, a rotational and cyclic planting regime between tobacco and *V. myuros* rows is enforced (Rotation + Intercropping group) (Zhang et al., 2020).

Treatment 2 (T2, rotation group): Conventional fertilization - based ridge cultivation is carried out, with 1.0 kg of compound fertilizer per row. At the end of September, green manure (300 g of rapeseed per plot) is broadcast - sown, and at the end of March, the green manure is turned under for soil improvement (Fang et al., 2016).

T3 (Treatment 3, cake fertilizer group): Ridge cultivation with conventional fertilization is practiced, with 1.0 kg of compound fertilizer and 1 kg of fermented cake fertilizer per row respectively.

### 2.2 Quantification of functional gene abundances

The PCR products were purified using magnetic bead purification. Samples were mixed in equidensity ratios based on the concentration of PCR products. After thorough mixing, the PCR products were detected and target bands were recovered (Fatima et al., 2014). Sequencing libraries were generated and indexes were added. The library was checked with Qubit and real-time PCR for quantification and bioanalyzer for size distribution detection. Quantified libraries were pooled and sequenced on Illumina platforms, according to effective library concentration and data amount required (Bokulich et al., 2013).

We employed a multi-region primer amplification strategy targeting bacterial 16S (V3/V4/V4-V5), archaeal 16S (ArcV4), and fungal ITS (ITS1/ITS2) regions using barcoded primers (e.g., 515F-806R for 16SV4). PCR reactions utilized Phusion High-Fidelity Master Mix with thermal cycling: 98°C/1 min initial denaturation, 30 cycles (98°C/10 s, 50°C/30 s, 72°C/30 s), and 72°C/5 min final extension. PCR products were purified via magnetic beads, pooled by concentration, and validated before library preparation. Libraries were quantified using Qubit and qPCR, then sequenced on Illumina platforms (Bokulich et al., 2013). Bioinformatics analysis included: 1) demultiplexing reads by barcode/primer trimming; 2) FLASH-based paired-end assembly into Raw Tags; 3) fastp-filtered Clean Tags; 4) chimera removal via SILVA (16S) and UNITE (ITS) database alignment using vsearch, generating Effective Tags for downstream analysis (Magoč and Salzberg, 2011).

We processed Effective Tags using the QIIME2 software: denoising was performed via DADA2 or deblur modules to generate Amplicon Sequence Variants (ASVs, default: DADA2). Species annotation was conducted in QIIME2, with Silva Database for 16S rRNA genes, UNITE Database for ITS regions, and the Micro_NT sub-library (extracted from NT database for archaea, fungi, viruses, and bacteria) for non-standard genomic regions. The primer sequences used for each region were listed in **Supplementary Table S1**.

### 2.3 Statistical analysis

Paired-end reads were merged using FLASH (v1.2.11, http://ccb.jhu.edu/software/FLASH/) (Magoč and Salzberg, 2011), a very fast and accurate analysis tool, which was designed to merge paired end reads when at least some of the reads overlap theread generated from the opposite end of the same DNA fragment, and the splicing sequences were called raw tags (Magoč and Salzberg, 2011). Quality filtering on the raw tags was performed using the fastp (v0.23.1) software to obtain high-quality Clean Tags (Bokulich et al., 2012). The tags were compared with the reference database [Silva database (16S), https://www.arb-silva.de/; Unite Database (ITS), https://unite.ut.ee/] to detect chimera sequences, and the effective tags were obtained by removing the chimera sequences with the vsearch package (v2.16.0) (https://github.com/torognes/vsearch) (Edgar et al., 2011). For the Effective Tags obtained previously, denoising was performed with DADA2 or deblur module in the QIIME2 software to obtain initial ASVs (Amplicon Sequence Variants) (default: DADA2). Species annotation was performed using QIIME2 software. For 16S, the annotation database is Silva Database, while for ITS, it is Unite Database. For the un- regular region, the default database is Micro_NT (a sub library obtained by extracting archaea, fungi, viruses, and bacteria from the NT) (Yan et al., 2019).

A series of statistical analyses which include Anosim, Adonis, Multi-response permutation procedure (MRPP), Simper, T-test, MetagenomeSeq and LEfSe, was performed to reveal the community structure differentiation. Anosim, Adonis and MRPP analyses are non-parametric tests that analyze the differences between high-dimensional data groups. They can test whether the differences between groups are significantly greater than the differences within the group, which can determine whether the grouping is meaningful. All of them were performed with vegan and ggplot2 package within R. Simper can reveal the contribution of each species to the differentiation between groups. Top 10 species were selected and presented on the graph. It was performed in R with Vegan package and ggplot2 package. MetagenomeSeq can showcase the species that display significant differences between groups. It was performed in R with metagenomeSeq package. LEfSe is widely used to discover biomarkers and it can reveal metagenomic characteristics. To achieve this, an exclusive package named lefse was utilized (Bachy et al., 2013).

To explore the symbiotic relationship between species and to reveal the environmental factor influence on the community structures, 2D and 3D network diagrams were drawn for visualization. Further analyses such as spearman correlation test, canonical correspondence analysis (CCA)/redundancy analysis (RDA) and dbRDA can be used to reflect the correlation between environmental factors and species abundance. All of these diagrams and analysis were completed in R.

Top 10 taxa of each sample at each taxonomic rank (Phylum, Class, Order, Family, Genus, Species) were selected to plot the relative abundance distribution histogram using the SVG function in Perl. We chose barplots of Phylum, Genus and Species levels for analysis.

In order to analyze the diversity, richness and uniformity of the communities in the sample, alpha diversity was calculated from 7 indices in QIIME2, including Observed_otus, Chao1, Shannon, Simpson, Dominance, Good’s coverage and Pielou_e. We chose Shannon and Chao1 for analysis.In order to evaluate the richness of microbial community and determine the appropriate sample size, the species accumulation boxplot can be used for visualize, which was performed with the vegan package in R software (Bokulich et al., 2018).

Simper can reveal the contribution of each species to the differentiation between groups. Top 10 species were selected and presented on the graph. It was performed in R with Vegan package and ggplot2 package.

### 2.4 Untargeted metabolomics

Metabolites were extracted from tissue (100 mg homogenized in liquid nitrogen), liquid (100 μL), cell/bacteria, or culture medium samples using prechilled 80% methanol, followed by ice incubation, centrifugation (15,000 g, 4°C), and dilution to 53% methanol prior to LC-MS/MS injection. UHPLC-MS/MS analysis utilized a Vanquish UHPLC system coupled with Orbitrap Q Exactive™ HF/HF-X mass spectrometer (Thermo Fisher) equipped with a Hypersil Gold column (100×2.1 mm, 1.9 μm) under a 12-min gradient (0.2 mL/min; mobile phase: 0.1% FA in water [A] and methanol [B]). Data processing via Compound Discoverer 3.3 included peak alignment, normalization (total spectral intensity), and metabolite identification using mzCloud, mzVault, and MassList databases. Statistical standardization (QC-based Coefficient of Variation filtering, CV < 30%) and formula-based normalization were applied. Metabolites were annotated via KEGG, HMDB, and LIPIDMaps databases. Multivariate analyses (PCA, PLS-DA) and univariate t-tests (VIP > 1, p < 0.05, FC ≥ 2 or ≤ 0.5) identified differential metabolites, visualized by volcano plots and heatmaps (z-score normalized). Metabolic pathway enrichment (x/n > y/N, p < 0.05) and Pearson correlation analysis (corrplot package) were performed using R/Python. Instrument parameters: 3.5 kV spray voltage, 320°C capillary, 35 psi sheath gas, 10 L/min aux gas, 350°C heater (Want et al., 2013).

### 2.5 Combined transcriptome and metabolome analysis

Microbiome analysis enables the identification of structural and abundance variations in microbial communities, along with functional predictions or annotations, while metabolomics directly reflects the functional interactions between microbial communities and their hosts. These two approaches are complementary and indispensable. Integrating “microbiomics” with “metabolomics” allows a deeper understanding of how environmental microbial communities influence host or environmental metabolic states through microbial metabolism and host co-metabolism. In this study, we performed metabolomic profiling and metagenomic sequencing on samples, followed by correlation analysis between differential metabolites and microbial taxa (Hess et al., 2011).

Spearman rank correlation analysis measures the association between two variables using the Spearman correlation coefficient, with rank correlation tests to determine statistical significance. Unlike Pearson correlation, which assumes normal distribution, Spearman correlation relies on rank statistics and imposes no assumptions on data distribution. The Spearman coefficient ranges from -1 to 1, where positive/negative values indicate positive/negative correlations, and larger absolute values denote stronger associations (|r| = 1 indicates perfect correlation). Correlations were computed using the cor function in R, and significance (p-values) was assessed via the corPvalueStudent function from the WGCNA package (Ilhan et al., 2017).

## 3 Results

### 3.1 Diversity of rhizosphere bacterial and fungal communities among the different crop rotation and fertilization

**Figure 1:**
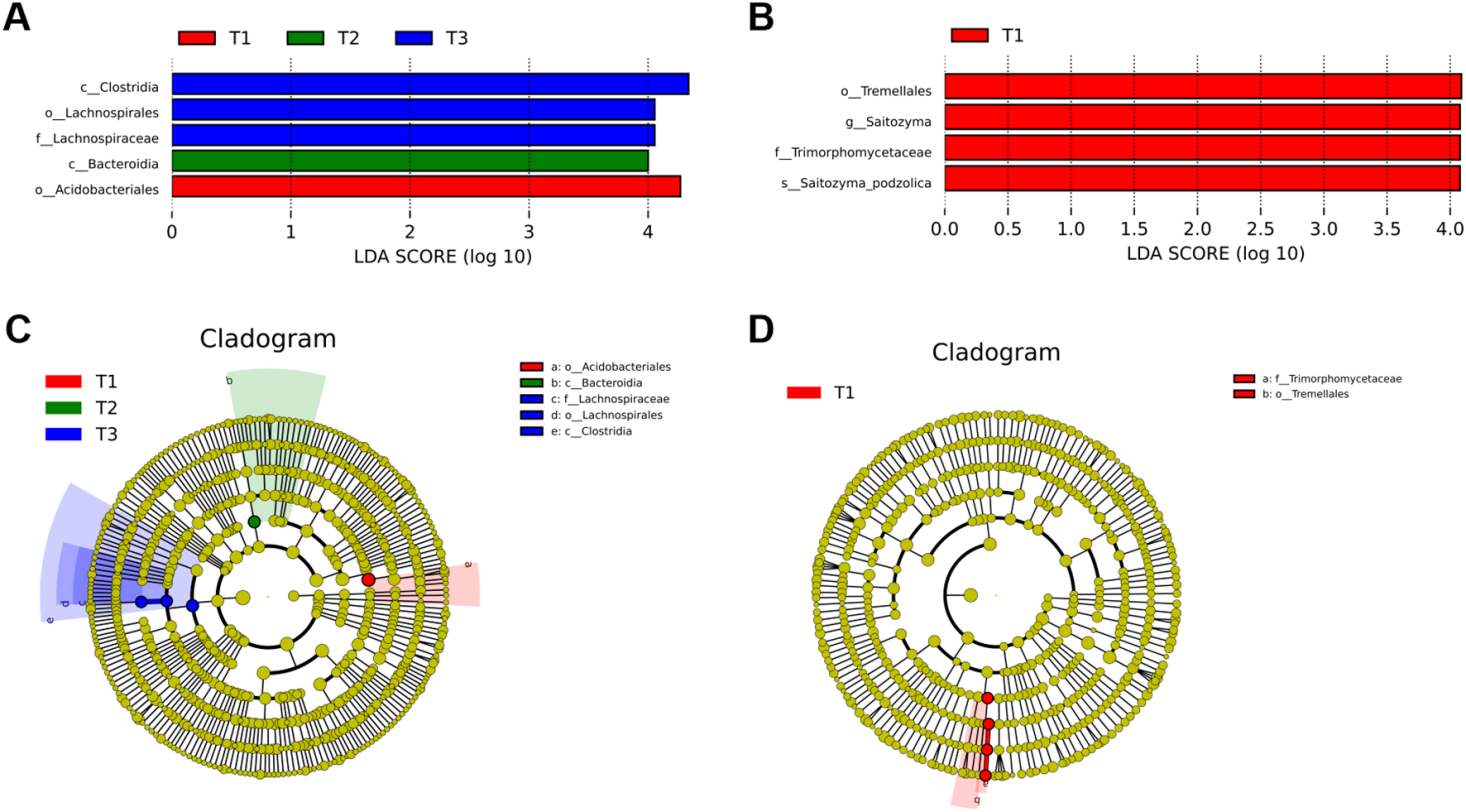
LEfSe analysis of microbial biomarkers across fertilization regimes. **(A)** LDA scores of bacterial biomarkers in T1 (red), T2 (green), and T3 (blue). **(B)** Fungal biomarkers with T1-specific enrichment. **(C)** Cladogram (phylum to family level) highlights T1-associated taxa (red), T2 (green), and T3 (blue); node size reflects relative abundance. **(D)** Phylogenetic clustering of T1-linked bacterial lineages (red gradient) and rare taxa (gray). Solid/dashed lines denote mean/median relative abundance; absent bars indicate undetectable biomarkers (LDA threshold >2, Kruskal-Wallis p < 0.05).

ANOSIM and Adonis analyses of 16S (**Supplementary Tables S2**, **S3**) and ITS (**Supplementary Tables S4**, **S5**) revealed a convergent pattern of the four experimental groups. LEfSe analysis identified treatment-specific microbial biomarkers with linear discriminant analysis scores exceeding 4.0 (**Figure 1**). Bacterial communities in CK were characterized by Acidobacteria and Bacteroidia (LDA 4.2-4.5), whereas T3 exhibited significant enrichment of Thermoanaerobacter (LDA 4.8) associated with lignocellulose degradation. T1 elevated plant-associated Sphingomonas (LDA 4.3) to 10.4% compared to 6.3% in CK, while T2 enriched nitrogen-cycling Rhodanobacter (LDA 4.1) to 8.7% versus 5.2% in CK. Differential selection of Burkholderia was observed between T1 (LDA 3.9) and T2 (LDA 2.7), reflecting manure type-driven niche specialization. Fungal communities in CK were dominated by Absidia (LDA 4.0), T3 exhibited saprophytic Mortierella dominance (LDA 4.5), and T2 uniquely hosted mycorrhizal Entrophospora (LDA 4.2), which was undetected in other treatments. Fungal assemblages demonstrated lower β-diversity than bacterial counterparts, with Ascomycota consistently constituting 72-76% of communities across all groups. Critical functional divergences emerged wherein T3 preferentially recruited carbon-metabolizing taxa (Thermoanaerobacter, Mortierella), contrasting CK’s Acidobacteria-Absidia profile, while T1 and T2 synergistically enriched nitrogen-cycling specialists (Sphingomonas, Rhodanobacter) and symbionts (Entrophospora in T2). Inter-treatment comparisons revealed T1’s selection for Burkholderia versus T2’s specialization toward Entrophospora, demonstrating crop-specific modulation of functional guilds. These results establish that fertilization strategies differentially reconfigure microbial consortia, with T3 enhancing organic matter mineralization capacity and T1/T2 promoting plant-microbe symbiotic networks.

**Figure 2:**
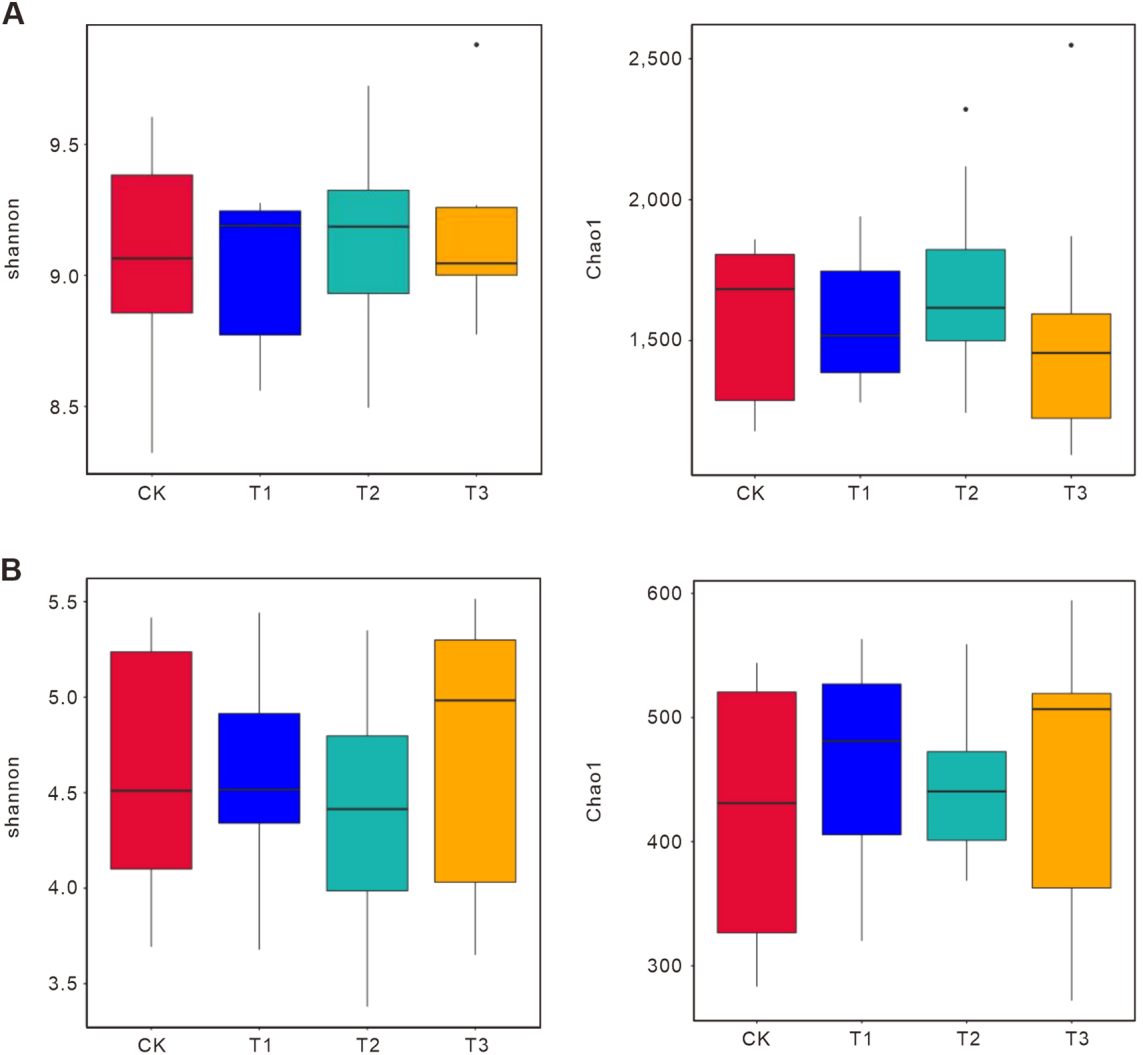
Alpha diversity indices (Shannon and Chao1) of bacterial and fungal communities across treatments. Alpha diversity indices of bacterial **(A)** and fungal **(B)** reflect species richness and evenness within samples. Higher index values indicate greater microbial community complexity. Statistical significance tests between groups can quickly identify groups with significantly increased or decreased diversity for further biological analysis. Boxplots visually compare diversity across groups. The Shannon index accounts for total taxa and their proportional abundances, with higher values indicating greater diversity and even species distribution. The Chao1 index estimates total species richness in a community, incorporating data from species with abundance 1 and 2 to better reflect low-abundance taxa. y-axis: index values; x-axis: treatment groups. “*” indicate statistically significant differences between corresponding groups. CK: Conventional fertilization. T1: Rotation + Intercropping group. T2: Rotation group. T3: Cake fertilizer group.

Furthermore, Alpha diversity indices (Shannon and Chao1) revealed distinct microbial responses (**Figure 2**). For 16S, T3 had the highest bacterial diversity (Shannon: 9.5; Chao1: 2500) compared to CK (Shannon: 9.0; Chao1: 1500), driven by fertilizer cake-derived organic matter (Jiang et al., 2022). T1 and T2 showed moderate increases (Shannon: 9.3; Chao1: 2000), suggesting selective enrichment of copiotrophic taxa. For ITS, T3 also had the highest fungal diversity (Shannon: 5.5; Chao1: 600) compared to CK (Shannon: 3.5; Chao1: 300), reflecting fertilizer cake-induced proliferation of rare taxa like Mortierellomycota (Su et al., 2022). T1 and T2 had intermediate diversity (Shannon: 5.0–4.0; Chao1: 500–400), with T1 favoring Glomeromycota and T2 promoting Basidiomycota. T3 significantly increased microbial diversity over CK (bacterial Chao1: +66.7%; fungal Chao1: +100%), while T1 and T2 showed moderate increases (bacterial Chao1: +33.3%; fungal Chao1: +66.7% in T1, +33.3% in T2). T1 prioritized symbiotic functions, while T2 favored decomposition. Between T1 and T2, bacterial diversity was similar (Chao1: 2000), but T2 had lower fungal diversity (Chao1: 400 vs. 500 in T1), likely due to rape’s recalcitrant lignin enriching specialized degraders.

**Figure 3:**
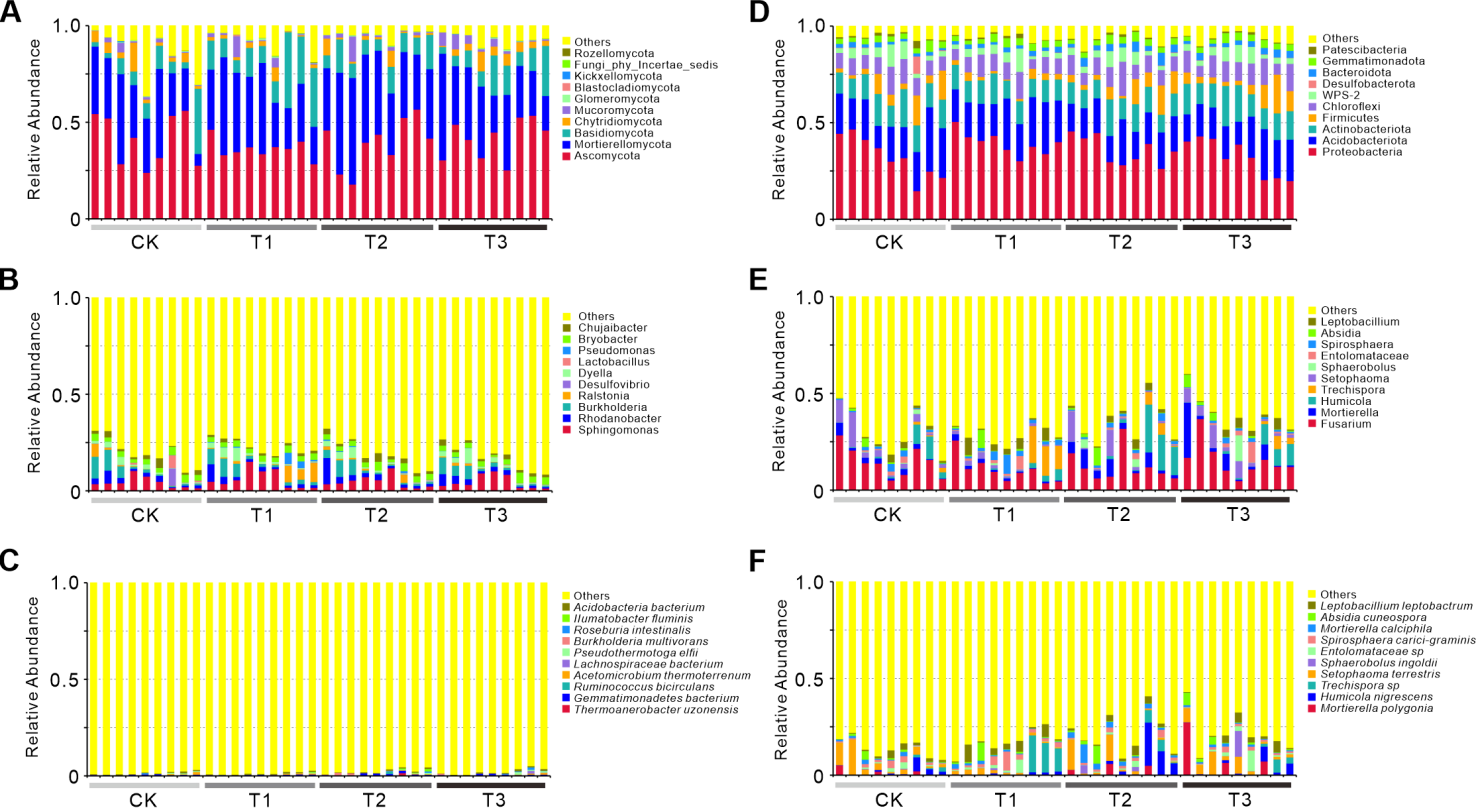
Relative abundance of bacterial and fungal communities at the phylum, genus, and species levels across treatments. Relative abundance of bacterial **(A, B, C)** and fungal **(D, E, F)** communities at the phylum **(A, D)**, genus **(B, E)**, and species **(C, F)** levels across treatments (CK, T1, T2, T3). Bar charts display the top 10 taxa by relative abundance at the phylum, genus, and species levels, with remaining taxa grouped as ‘Others’. Colors distinguish microbial taxa, while the x-axis indicates sample names and the y-axis shows relative abundance (%). Each bar aggregates low-abundance species into the ‘Others’ category, calculated by summing relative abundances beyond the top 10 ranked taxa. CK: Conventional fertilization. T1: Rotation + Intercropping group. T2: Rotation group. T3: Cake fertilizer group.

The phylum-level composition of bacterial (16S) and fungal (ITS) communities showed distinct responses to treatments (**Figure 3**). In 16S analysis, CK was dominated by Proteobacteria and Actinobacteria. T3 had increased Firmicutes and Bacteroidota, linked to enhanced organic matter decomposition from high-carbon fertilizer cake (Rampadarath et al., 2018). T1 showed reduced Proteobacteria but elevated Acidobacteria, likely due to *V. myuros*-induced soil acidification (Kalam et al., 2020). T2 uniquely enriched Chloroflexi, potentially tied to recalcitrant lignin from rape residues (Atiwesh et al., 2022). For ITS analysis, Ascomycota dominated all groups. T3 had reduced Basidiomycota, possibly due to fertilizer cake suppressing ligninolytic fungi (Atiwesh et al., 2022). T1 showed a striking increase in Glomeromycota, consistent with *V. myuros*-enhanced arbuscular mycorrhizal symbiosis (Sawers et al., 2018). T2 had higher Basidiomycota, likely supporting rape lignocellulose degradation (Atiwesh et al., 2022). Bacterial communities in T1 favored oligotrophic Acidobacteria, while T2 enriched copiotrophic Chloroflexi. Fungal communities in T1 were dominated by Glomeromycota, contrasting with Basidiomycota specialization in T2, reflecting differential carbon utilization between *V. myuros* and rape cultivation (Zhou et al., 2018). These results highlight treatment-specific impacts: T3 enhanced decomposition, T1 promoted symbiosis, and T2 favored recalcitrant carbon degradation.

Barplots of genus level demonstrated distinct treatment-specific microbial responses. T3 significantly enriched Fusarium, with bacterial abundance reaching 14.8% compared to 3.2% in control (CK) and fungal abundance at 18.3% versus 6.5% in CK, alongside Humicola at 12.1% relative to 3.9% in CK, indicating a selective enhancement of carbon-degrading taxa. T1 elevated Sphingomonas to 10.4% from 6.3% in CK and increased Entolomataceae to 9.4%, while T2 upregulated Rhodanobacter to 8.7% compared to 5.2% in CK and Sporophila to 10.2%, reflecting functional niche differentiation driven by green manure specificity. Bacterial communities exhibited stronger treatment-associated divergence, as exemplified by Burkholderia abundance (7.1% in T1 versus 4.9% in T2), whereas fungal communities maintained structural consistency with Ascomycota persistently dominating across all treatments at 72–76% relative abundance.

Species-level barplots demonstrated distinct treatment effects. Bacterial communities in CK predominantly harbored Acidobacteria and Roseburia, whereas T3 exhibited significant enrichment of thermophilic Thermoanaerobacter taxa linked to carbon metabolism. T1 elevated plant growth-promoting Sphingomonas to 10.4% compared to 6.3% in CK, while T2 enhanced nitrogen-cycling Rhodanobacter to 8.7% versus 5.2% in CK, concurrent with differential Burkholderia abundance between T1 (7.1%) and T2 (4.9%). Fungal profiles revealed CK dominated by Absidia, T3 characterized by saprophytic Mortierella, and T2 uniquely hosting mycorrhizal Entrophospora at 9.4% abundance, with fungal assemblages displaying reduced inter-treatment variability relative to bacterial counterparts as Ascomycota consistently constituted 72–76% of total communities. Critical functional divergences emerged wherein T3 prioritized carbon-metabolizing taxa, whereas T1 and T2 enriched nitrogen cyclers and symbionts, reflecting crop-specific microbial selection dynamics.

### 3.2 PCA and KEGG pathway analysis of tobacco rhizosphere soil metabolites

**Figure 4:**
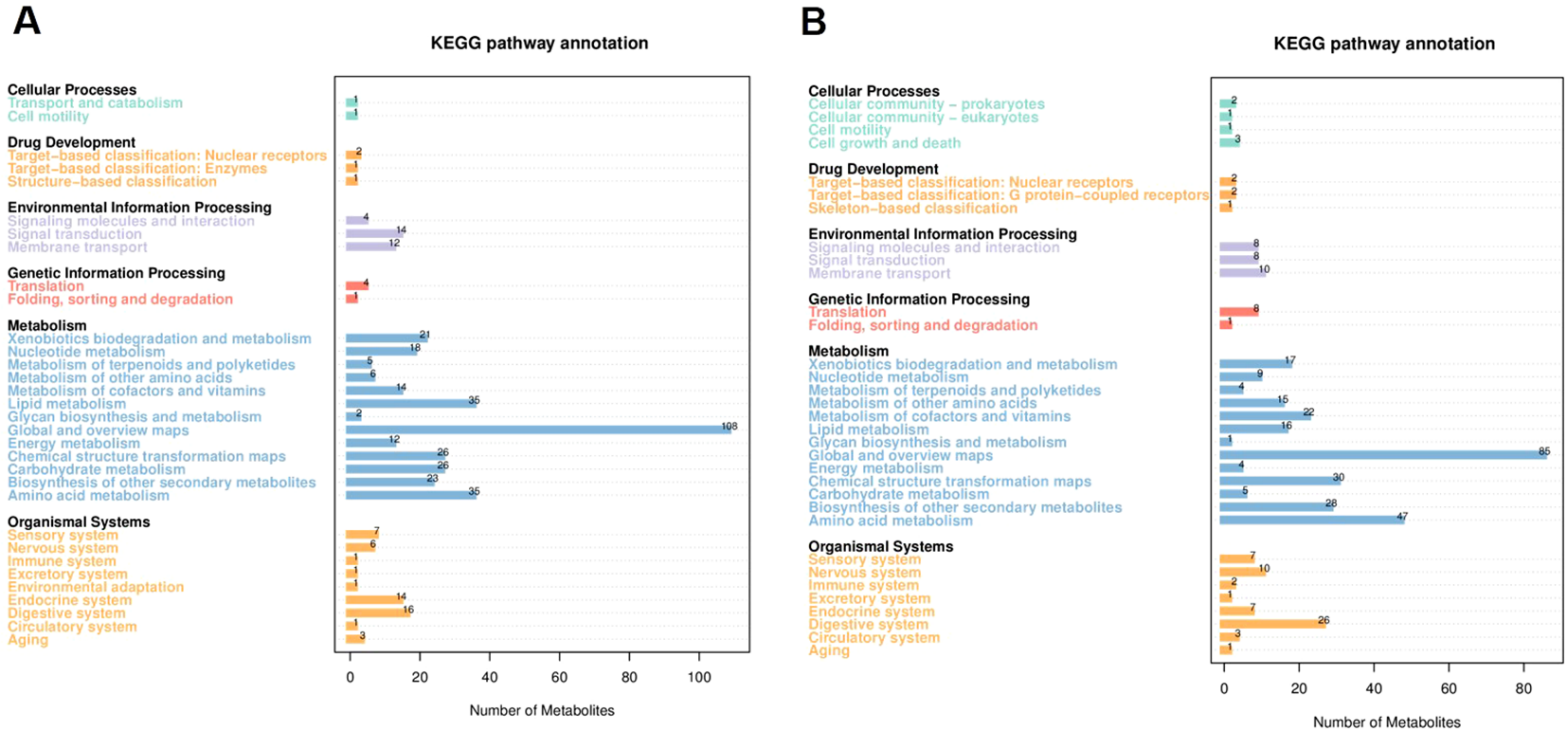
The annotation of negative and positive metabolites within the KEGG pathways. The horizontal coordinates negative **(A)** and positive **(B)** represent the number of metabolites, and the vertical coordinates represent the annotated KEGG pathways. This figure provides a comprehensive overview of the distribution of metabolites across various KEGG pathways at the secondary classification level. Each bar in the bar chart corresponds to a specific secondary-classified KEGG pathway, with the height of the bar indicating the number of metabolites associated with that particular pathway.

Principal component analysis (PCA) of four experimental and QC samples revealed distinct scores for PC1 and PC2 (**Supplementary Figure S1**). The concentration of QC samples indicates that the test process was highly stable (Bokulich et al., 2013). Kyoto Encyclopedia of Genes and Genomes (KEGG) pathway enrichment analysis of the annotated metabolites identified six major functional categories (first-level classification): Cellular processes, drug development, environmental information processing, genetic information processing, metabolism, and organismal systems. Among the secondary classification units, the top three categories were global and overview maps, amino acid metabolism, and lipid metabolism (**Figure 4**). These pathways, particularly those related to global and overview maps, amino acid metabolism, and lipid metabolism, are crucial for understanding the tobacco soil metabolome (Shi et al., 2024). The global and overview maps pathway provides a comprehensive view of tobacco soil metabolism through the KEGG atlas, which includes global metabolic maps, data visualization, and auxiliary pathway analysis (Qiao et al., 2023). This helps in understanding the interrelationships, changes, and mechanisms of tobacco soil metabolites. Microorganisms influence amino acid content and composition in the soil by affecting amino acid uptake and utilization, which is further influenced by microbial community structure, soil environmental factors (such as pH and carbon content), and the feedback from metabolic products (Babalola et al., 2021).

### 3.3 Microbial co-occurrence networks reveal treatment-specific interaction patterns

**Figure 5:**
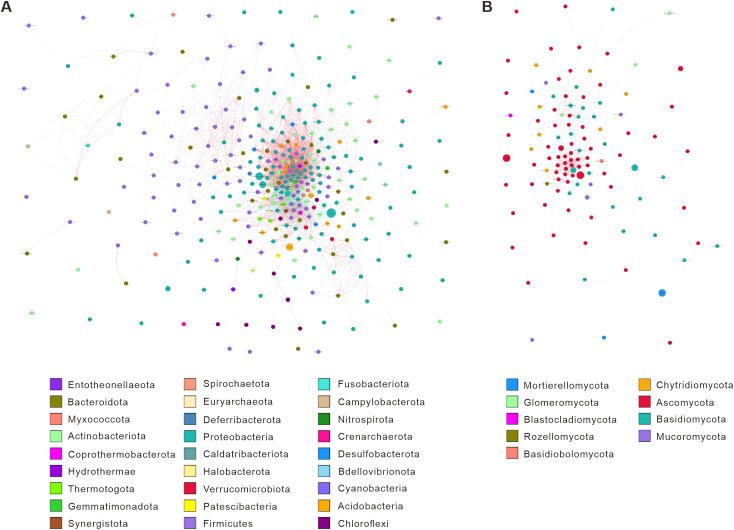
Microbial interaction networks based on Spearman correlations for 16S and ITS sequencing across treatments. The network diagram was generated by calculating Spearman correlation coefficients for 16S **(A)** and ITS **(B)** sequencing across all samples to form a species correlation matrix. Filtering conditions were applied: removing correlations with coefficients <0.6; excluding self-connections; discarding links where node abundance was <0.005%. Nodes represent genera (size indicates average relative abundance; colors denote phylum-level taxonomy). Edges reflect species interactions: thickness corresponds to the absolute correlation coefficient, while colors indicate positive (red) or negative (blue) correlations.

Spearman-based co-occurrence networks revealed treatment-specific modulation of bacterial (16S) and fungal (ITS) interactions (**Figure 5**). For 16S data, T3 exhibited the highest connectivity (edges: 68 vs. CK: 41), dominated by Firmicutes and Bacteroidota with 78% positive correlations, indicating fertilizer cake-enhanced synergistic decomposition. CK showed sparse connections among Proteobacteria, while T1 formed modular clusters between Acidobacteria and Proteobacteria (edges: 55), reflecting niche partitioning driven by *V. myuros*. T2 displayed fragmented networks centered on Chloroflexi, with 49% negative correlations, suggesting lignin-driven competitive exclusion.

For ITS data, T3 featured reduced Basidiomycota interactions but strengthened positive correlations between Mortierellomycota and Ascomycota (edges: 45 vs. CK: 28), aligning with fertilizer cake-mediated lipid metabolism (Fallah et al., 2023). T1 showed Glomeromycota hubs (degree: 8) with 89% positive correlations, supporting *V. myuros*-induced mycorrhizal symbiosis, while T2 centered on Basidiomycota-Chytridiomycota co-dependencies (edges: 32), driven by rape lignocellulose degradation.

Comparisons between treatments further highlighted distinct microbial dynamics. T3 bacterial edge density increased by 65.9%, and fungal positive correlations rose by 24.1% compared to CK, underscoring fertilizer cake’s role in fostering cooperative guilds. T1 exhibited higher bacterial modularity (0.46 vs. CK: 0.32) and expanded fungal Glomeromycota nodes (6 vs. CK: 2), reflecting intercropping-mediated mutualism (Wang et al., 2016). In contrast, T2 showed dominant bacterial negative correlations (48.7% vs. CK: 31.5%) and fungal Basidiomycota specialization, indicating rape residue-driven competition. Between T1 and T2, T1 prioritized Acidobacteria symbiosis (edges: 14 vs. T2: 5), while T2 emphasized Basidiomycota-Chytridiomycota decomposer co-occurrence (edges: 9 vs. T1: 2). These results demonstrate that carbon source quality (fertilizer cake vs. plant residues) and planting regimes (intercropping vs. rotation) reconfigure microbial interaction strategies, fostering distinct functional guilds and ecological dynamics.

### 3.4 Lipid metabolite profiling in tobacco rhizosphere soil

**Figure 6:**
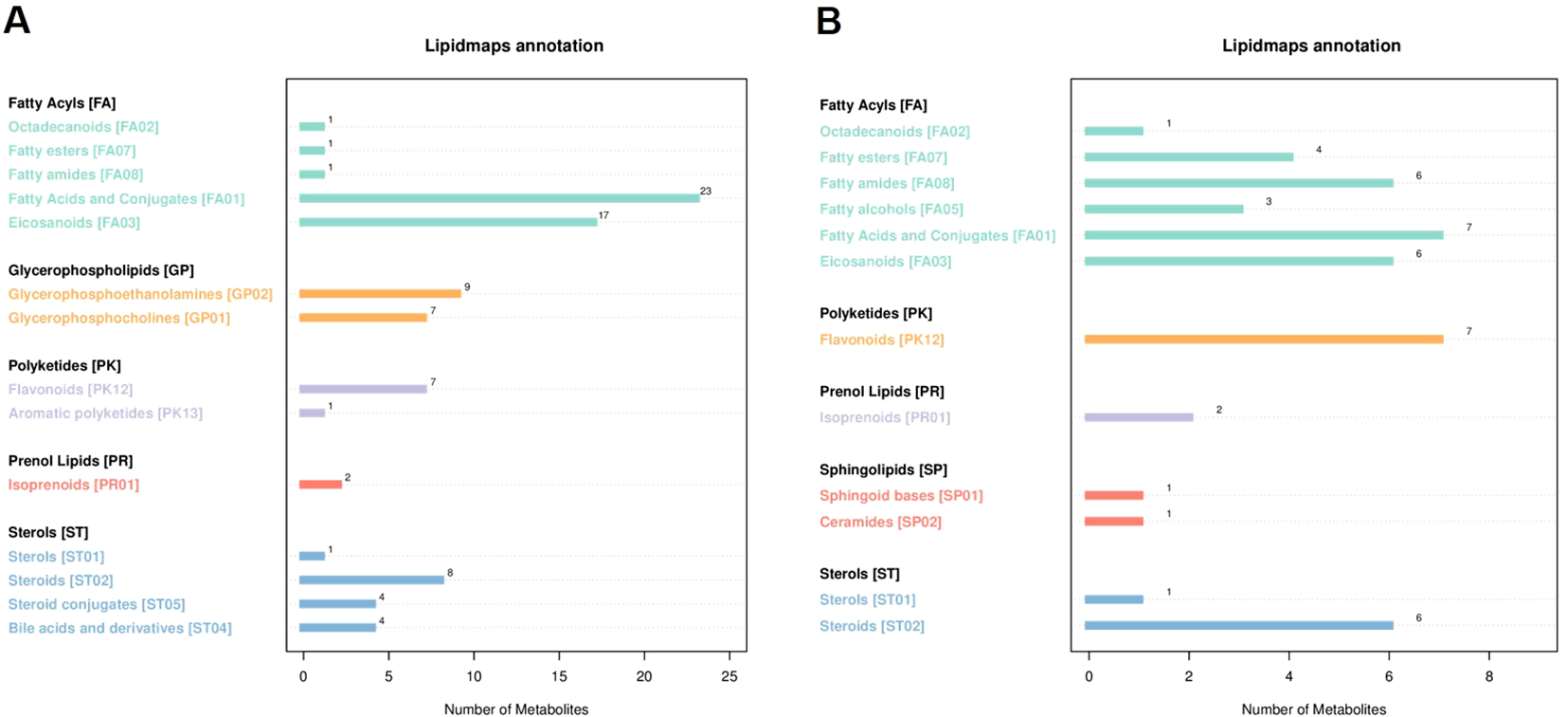
The number of metabolites, and the ordinate represents the lipid classification of LIPID MAPS annotated. **(A)** negative lipid metabolites. **(B)** positive lipid metabolites. This figure shows the number of (lipid) metabolites corresponding to the main level classification (Main Class) under the eight major lipid classifications (Category) in LIPID MAPS. The x-axis indicates the number of metabolites, while the y-axis lists the lipid classes. The bar lengths in each panel reflect the metabolite counts for each lipid class.

Lipid metabolites were annotated using the lipid maps database, revealing a total of 86 down-regulated lipid metabolites (**Figure 6**). The top three were 23 types of Fatty Acids and Conjugates [FA01], 17 types of Eicosanoids [FA03], and 9 types of Glycerophosphoethanolamines [GP02]. Additionally, 45 up-regulated lipid metabolites were identified, with the top three being 7 types of fatty acids and conjugates [FA01], 7 types of flavonoids [PK12], and 6 types of steroids [ST02].

Fatty acids and conjugates [FA01] can indirectly influence tobacco growth by affecting the structure of soil microbial communities and participating in nutrient cycling. Eicosanoids [FA03] serve as plant-microbe signaling molecules in tobacco soil. They facilitate the aggregation of beneficial microorganisms and play a critical role in tobacco defense mechanisms by activating defense gene expression. In conclusion, lipid metabolite profiling in tobacco soil revealed significant changes, with down-regulated fatty acids and eicosanoids influencing microbial community structure, nutrient cycling, and plant defense, while up-regulated metabolites, such as flavonoids and steroids, further contributed to soil ecosystem stability, providing a clear overview of the lipidomic changes that underpin soil health and tobacco growth.

### 3.5 Pathway enrichment analysis of differential metabolites

**Figure 7:**
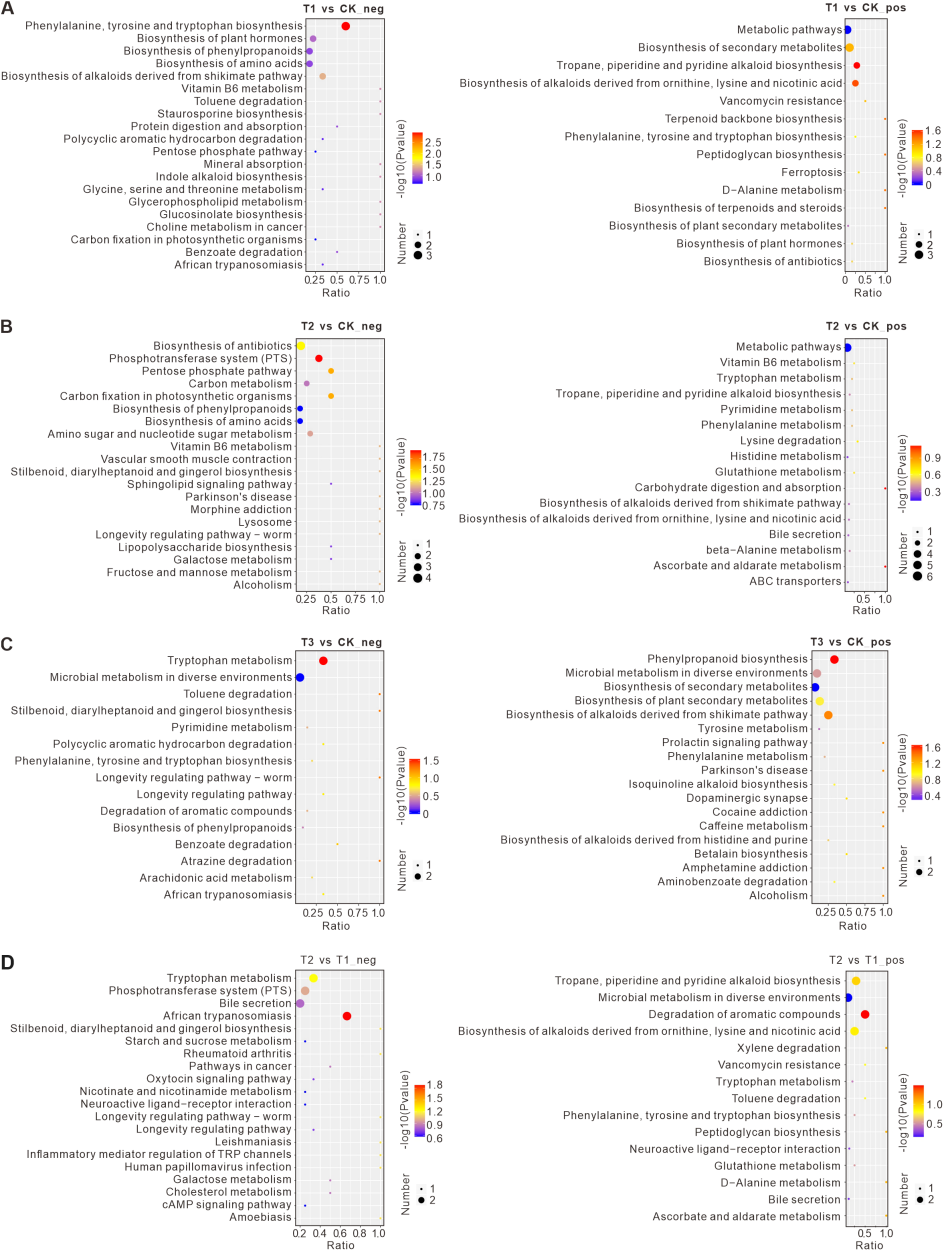
The top 20 pathways according to the KEGG enrichment results. **(A)** Negative and positive metabolites in the comparison between T1 and CK. **(B)** Negative and positive metabolites in the comparison between T2 and CK. **(C)** Negative and positive metabolites in the comparison between T1 and T2. **(D)** Negative and positive metabolites in the comparison between T3 and CK. The abscissa is x/y (the number of differential metabolites in the corresponding metabolic pathway/the total number of metabolites identified in the pathway). The larger the value, the higher the enrichment degree of differential metabolites in the pathway. The ordinate is the name of the KEGG pathway. The color of the point represents the P-value of the hypergeometric test. The smaller the value, the greater the reliability of the test and the more statistically significant. The size of the point represents the number of differential metabolites in the corresponding pathway. The larger the size, the more differential metabolites there are in the pathway. CK: Conventional fertilization, T1: Rotation + Intercropping group, T2: Rotation group, T3: Cake fertilizer group.

To investigate the potential effects of differential metabolites, pathway enrichment analysis was performed using the KEGG database (**Figure 7**). Compared to the control (CK), T1 treatment resulted in significantly down-regulated metabolites enriched in pathways such as the biosynthesis of phenylpropanoids, biosynthesis of amino acids, and the biosynthesis of alkaloids derived from the shikimate pathway. In T2 treatment, down-regulated metabolites were enriched in the biosynthesis of phenylpropanoids, biosynthesis of amino acids, and carbon metabolism. The changes in phenylpropanoid and amino acid biosynthesis were similar to those observed in T1. For T3 treatment, down-regulated metabolites were significantly enriched in pathways such as tryptophan metabolism and microbial metabolism in diverse environments. Up-regulated metabolites in T3 were enriched in pathways related to microbial metabolism, biosynthesis of secondary metabolites, and plant secondary metabolite biosynthesis.

To explore the differences of soil metabolites in different rotation groups, we also compared T1 and T2. Compared to T1, T2 treatment showed significant down-regulation of metabolites enriched in pathways such as Tryptophan Metabolism, the Phosphotransferase System (PTS), and Bile Secretion. Up-regulated metabolites in T2 were enriched in pathways such as Tropane, Piperidine, and Pyridine Alkaloid Biosynthesis, Microbial Metabolism in Diverse Environments, and Alkaloid Biosynthesis from Ornithine, Lysine, and Nicotinic Acid.

The total differential metabolite heatmap visually reveals the impact of different fertilization treatments on soil microbial-metabolic interaction networks (**Supplementary Figure S2**). In T1 treatment, 37 metabolites were significantly down-regulated compared to CK, including 2’-Deoxycytidine 5’-diphosphate (dCDP), 3-Hydroxybenzoic acid, Pyrithioxin, and Sodium cholate. Meanwhile, 67 metabolites were up-regulated, including 1,3-Dihydro-1,3,3-trimethyl-2H-indol-2-ylidene acetaldehyde, N-Acetyl-DL-phenylalanine, and 2-Isobutyl-3-methoxypyrazine.

In T2 treatment, 35 metabolites showed significant down-regulation compared to CK, including 1-(4-Methoxyphenyl)-2-propanone, 16-Hydroxyhexadecanoic acid, and 4-Nitrophenol. In contrast, 31 metabolites were up-regulated, including 5-phenyl-2,3-dihydro-1H-1,4-benzodiazepin-2-one, Isohomovanillic acid, and Kaempferol.

In T3 treatment, 24 metabolites were significantly down-regulated compared to CK, including Hexadecanedioic acid, Delta-Tridecalactone, and Glycocholic acid. Additionally, 39 metabolites were up-regulated, including 4-(3-methoxy-5,6-dihydrobenzo[c]acridin-7-yl) morpholine, 1,4-dihydroxyheptadec-16-en-2-ylacetate, and Pyridoxamine.

When comparing T2 with T1, 48 metabolites were significantly down-regulated in T2, including 1-(4-Methoxyphenyl)-2-propanone, 16-Hydroxyhexadecanoic acid, and N-Formylkynurenine. Conversely, 72 metabolites were up-regulated in T2, including 1,3-Dihydro-1,3,3-trimethyl-2H-indol-2-ylidene acetaldehyde, N-Acetyl-DL-phenylalanine, and (+/-)5(6)-EET Ethanolamide. Overall, the analysis revealed that different tobacco planting treatments significantly affected key metabolic pathways, including phenylpropanoid and amino acid biosynthesis, carbon metabolism, and microbial interactions, highlighting the functional shifts caused by different agricultural practices.

### 3.6 Gene abundance in soil microorganisms across treatments using 16S sequencing analysis

**Figure 8:**
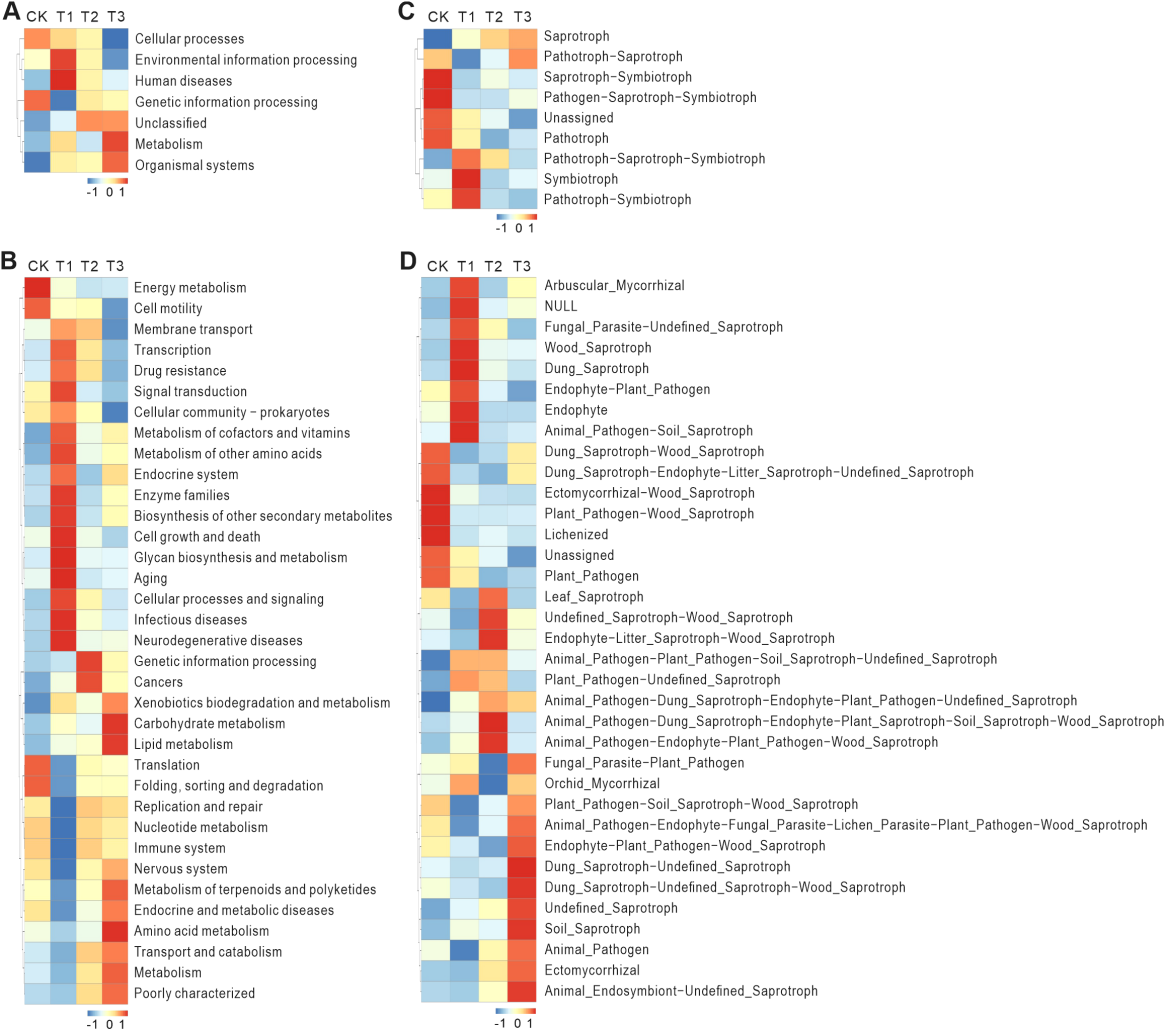
16S and ITS sequencing predicts functional annotation clustering heatmaps, displaying the same species heatmap. This heatmap is completed using Tax4Fun and FunGuild. Tax4Fun is an R package based on the 16S Silva database for functional prediction of environmental samples such as gut and soil. Based on the functional annotations and abundance information of samples in the database, the top 35 functions in terms of abundance and their abundance information in each sample are selected to draw the heatmap, and clustering is performed from different functional levels. FunGuild is an amplicon analysis based on ITS that can obtain the classification and abundance information of fungal species present in the environment, and can correspond to the ecological functions of fungi based on their species classification. Based on the functional annotations and abundance information of samples in the database, the top 35 functions in terms of abundance and their abundance information in each experimental group are selected to draw the heatmap, and clustering is performed from the perspective of functional differences. **(A)** The functional prediction analysis results of Level1 database. **(B)** The functional prediction analysis results of Level2 database. **(C)** The functional prediction analysis results of the mode database. **(D)** The functional prediction analysis results of the guild database. Horizontally represents experimental groups (CK: Conventional fertilization, T1: Rotation + Intercropping group, T2: Rotation group, T3: Cake fertilizer group), and vertically represents functional annotation information, with the clustering tree on the left side of the heatmap being the functional clustering tree; the corresponding values in the heatmap are the Z-scores obtained after standardizing the relative abundance of each row of functions, that is, the Z-score of a sample in a certain category is the difference between the relative abundance of the sample in that category and the average relative abundance of all samples in that category, divided by the standard deviation of all samples in that category.

The PCA plots of the four different experimental groups for 16S sequencing show that the experimental results of each group are relatively clustered, indicating that the experimental results are relatively stable (**Supplementary Figure S3**). The cluster heatmap from 16S sequencing of soil microorganisms revealed distinct differences in gene abundance across treatments (**Figure 8**). Compared to the CK, T1 treatment showed higher abundance of genes related to human diseases and environmental information processing (Level 1), such as transcription, signal transduction, and infectious and neurodegenerative diseases (Level 2). In T2, genes associated with genetic information processing and cancers (Level 2) showed higher abundance. Genetic information processing includes key cellular processes such as DNA replication, transcription, and translation. The elevated abundance of these genes suggests that soil microorganisms in T2 are actively replicating and expressing their genetic materials to adapt to the environmental conditions. In T3, genes related to metabolism and organismal systems (Level 1), including Metabolism of terpenoids and polyketides and amino acid metabolism (Level 2), were more abundant. Metabolism is a fundamental process for maintaining life, and the high abundance of metabolism-related genes in T3 indicates that the microorganisms are actively engaged in substance metabolism, obtaining energy and nutrients from the environment (McClure et al., 2020).

To sum up, 16S sequencing analysis revealed that different tobacco planting treatments influenced microbial gene abundance, with T1 showing increased transcription and signal transduction activity, T2 promoting genetic information processing, and T3 enhancing metabolic pathways related to nutrient acquisition.

### 3.7 ITS sequencing results of soil microorganisms across treatments

The PCA plots of the four distinct experimental groups for ITS sequencing demonstrate that the experimental outcomes of each group are relatively clustered, suggesting relatively stable experimental results (**Supplementary Figure S4**). The ITS sequencing results revealed significant differences in gene abundance across treatments (**Figure 8**). Compared to the CK, T1 treatment showed higher abundance of genes related to arbuscular mycorrhizal fungi, fungal parasites, undelined saprotrophs, wood saprotrophs, dung saprotrophs, endophytes, and plant pathogens. In T2, genes associated with undefined saprotrophs, wood saprotrophs, endophytes, and animal pathogens showed higher abundance. In T3, genes related to dung saprotrophs, undefined saprotrophs, and animal endosymbionts were more abundant.

In brief, the ITS sequencing analysis revealed distinct shifts in fungal gene abundance across treatments, with T1 showing increased levels of arbuscular mycorrhizal fungi and saprotrophic fungi, T2 enriching for wood saprotrophs and animal pathogens, and T3 promoting dung saprotrophs and animal symbionts, reflecting complex interactions between fungi, plants, and soil conditions.

### 3.8 Link between microbes and metabolites

**Figure 9:**
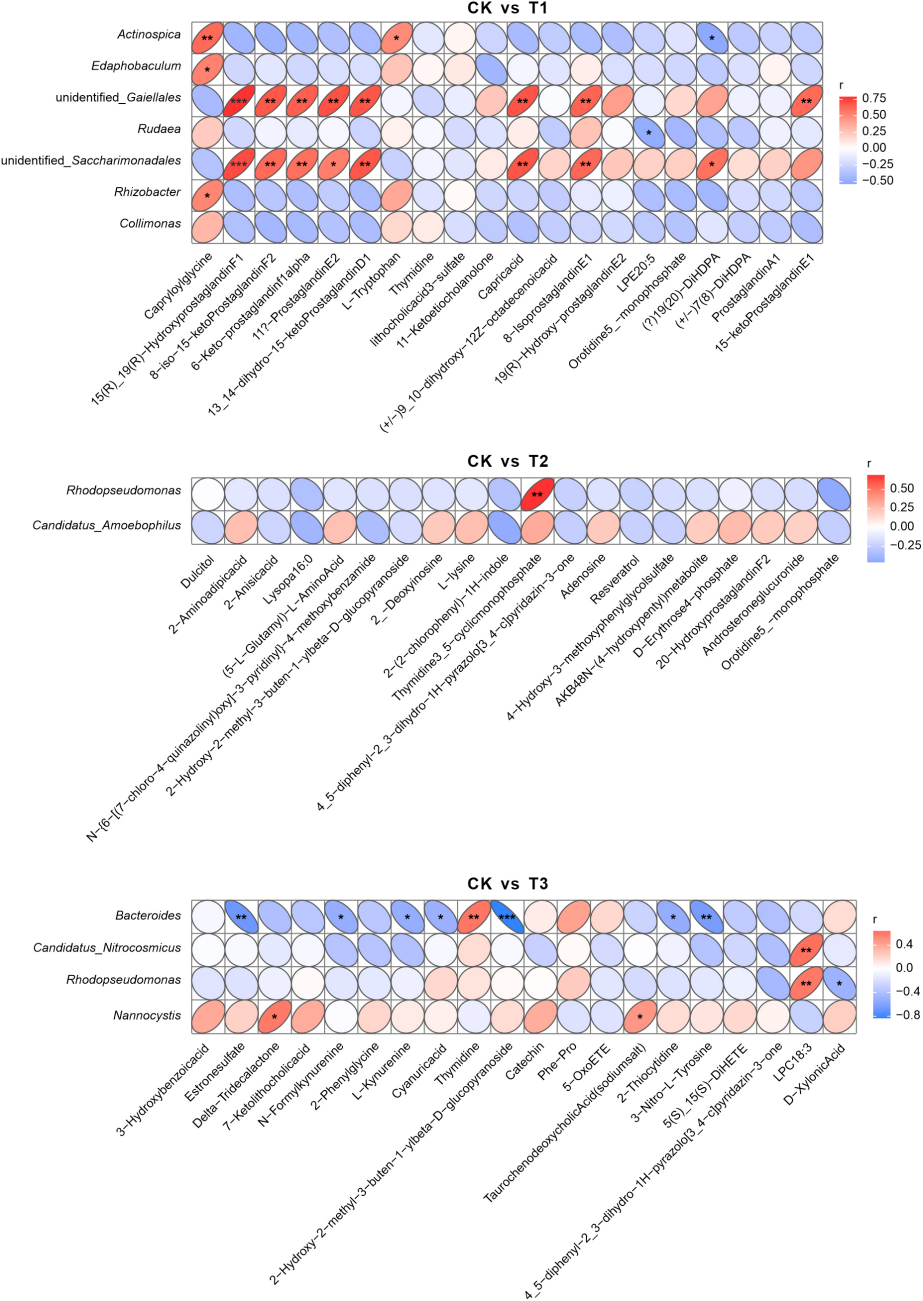
Heatmaps of Spearman correlations between microbial taxa and VIP-selected differential metabolites. The heatmaps demonstrate microbial genera/species (y-axis) versus metabolites (x-axis) across pairwise comparisons: CK vs T1, CK vs T2, and CK vs T3. Red/blue ellipses indicate positive/negative correlations (|r| ≥ 0.25, p < 0.05), with ellipse thinness inversely proportional to |r| magnitude. Blank cells denote non-significant associations (p ≥ 0.05). CK: Conventional fertilization. T1: Rotation + Intercropping group. T2: Rotation group. T3: Cake fertilizer group. *P<0.05; **P<0.01; ***P<0.001.

For combined 16S sequencing and metabolome analysis,the heatmap delineates significant Spearman correlations between microbial taxa and the top 20 variable importance in projection (VIP)-ranked metabolites across fertilization regimes (**Figure 9**). In comparative analyses between CK and T1, Actinospica belonging to Actinobacteria demonstrated a robust positive correlation with FR01.1801-Hydrogen, a carbon-nitrogen cycling-associated compound, exhibiting a correlation coefficient of 0.75 at a significance level of p < 0.05. This aligns with the role of T1 in enhancing organic matter turnover. Conversely, Rudena from Proteobacteria displayed a negative correlation with Copoxyoriso(a), a phenolic derivative, showing a coefficient of -0.50 (p < 0.05), indicative of suppressed oxidative stress-related metabolite synthesis under T1.Comparisons between CK and T2 revealed moderate positive correlations (r = 0.50, p < 0.05) between Phodopsseudomonas (Proteobacteria) and 2-Antropid(Ri), a nitrogen-containing alkaloid, consistent with T2 promoting nitrogen-metabolizing taxa through rape rotation. In CK versus T3 analyses, Candidatus_Attracolbidon exhibited a weak negative association (r = -0.40, p < 0.05) with D-Xivlenol, a lignin-derived compound, suggesting fermented cake amendments may attenuate lignocellulose degradation activity.These results demonstrate that T1 and T2 strengthen microbial-metabolite interactions associated with nutrient cycling, whereas T3 fosters taxon-specific but functionally weaker associations, reflecting divergent mechanisms by which organic amendments reconfigure rhizosphere metabolic networks.

For combined ITS sequencing and metabolome analysis, the heatmap delineates significant Spearman correlations between fungal taxa and the top 20 variable importance in projection (VIP)-ranked metabolites across fertilization regimes (**Supplementary Figure S5**). Comparative analysis between CK and T1 revealed that the fungal genus Saitozyma exhibited a robust positive correlation (r = 0.50, p < 0.05) with prostaglandin F1, a plant hormone-like metabolite implicated in stress signaling pathways, consistent with T1 promoting stress-responsive fungal symbionts. Conversely, Nectria demonstrated a negative correlation (r = -0.25, p < 0.05) with 8-iso-15-ketoProstaglandin F1, an oxidative stress-associated prostaglandin derivative, indicative of T1-mediated suppression of fungal oxidative stress pathways.In CK versus T2 comparisons, the taxon Oplidiomycota_gen_incertae_sedis displayed moderate positive correlations with resveratrol (r = 0.40, p < 0.05), a phytoalexin exhibiting antioxidant properties, alongside negative correlations with androsterone glucuronide (r = -0.40, p < 0.05), a steroid metabolite. This dual correlation pattern suggests T2 preferentially enriches fungal taxa involved in secondary metabolite biosynthesis while reducing steroid accumulation.These results demonstrate that T1 strengthens fungal interactions with stress-related metabolic networks, whereas T2 drives fungal community specialization toward antioxidant synthesis, reflecting distinct functional adaptation mechanisms to organic amendment strategies.

## 4 Discussion

This study presents comprehensive insights into the effects of different tobacco planting systems on soil metabolites, microbial communities, and their functional roles in the rhizosphere. The results suggest that crop rotation and intercropping with species such as *V. myuros* and rape can significantly alter soil metabolite profiles and microbial diversity, with important implications for soil health, plant growth, and pest management (Liu et al., 2024b).

PCA and KEGG pathway analyses revealed notable shifts in tobacco soil metabolomes under different treatments (Magoč and Salzberg, 2011). The pathways related to amino acid metabolism, lipid metabolism, and global metabolic maps were particularly prominent, highlighting the complex biochemical interconnections that govern nutrient cycling and plant health (Sun et al., 2023b). Fatty acids and conjugates [FA01] down-regulated in T1 were particularly linked to changes in microbial cell membrane integrity, which may have influenced microbial diversity and nutrient cycling processes. Changes in these metabolites can alter microbial cell membrane characteristics, thus impacting microbial populations (Wu et al., 2024a). Their metabolic products contribute to the cycling of key elements, such as carbon and nitrogen, ultimately affecting the nutrient availability and growth of tobacco plants (Zhang et al., 2024). Up-regulation of flavonoids and steroids in T1 may have contributed to improved soil stability and plant defense mechanisms, underscoring the intricate feedback between tobacco plants and their microbial environment (Zhuang et al., 2024). Eicosanoids [FA03] serve as plant-microbe signaling molecules in tobacco soil. They facilitate the aggregation of beneficial microorganisms and play a critical role in tobacco defense mechanisms by activating defense gene expression. These metabolites also contribute to soil ecosystem stability by reducing the accumulation of pathogenic bacteria and maintaining microbial diversity (Fallah et al., 2023). Changes in tryptophan metabolism are linked to neurotransmitter synthesis and physiological regulation, while the PTS plays a key role in substance transport and energy metabolism (Wang et al., 2023). Alterations in bile secretion pathways may affect substance metabolism and detoxification (Liu et al., 2024c).

In addition, lipid metabolism pathways, which interact with other pathways such as fatty acid metabolism, PPAR signaling, and glyceride metabolism, also play a significant role in tobacco soil metabolomics (Lin et al., 2022). These pathways impact the soil metabolites by altering microbial community structure, regulating gene expression, and participating in signal transduction (Kumawat et al., 2022). Moreover, continuous tobacco cropping and the use of different tobacco varieties affected rhizosphere microorganisms and metabolites, indirectly linking lipid metabolism pathways to other biological processes (Chen et al., 2024b). Altogether, these findings emphasize the complex relationship between tobacco cultivation, soil metabolites, and microbial diversity.Phenylpropanoids play critical roles in plant growth, stress resistance, and development (Wang et al., 2020). Amino acids, as fundamental building blocks of proteins, are crucial for protein synthesis and metabolism, influencing overall physiological functions (Lei et al., 2017). The alkaloid biosynthesis pathway, derived from the shikimate pathway, is involved in plant defense mechanisms and physiological regulation. Up-regulated metabolites in T1 treatment were enriched in pathways including metabolic pathways, biosynthesis of secondary metabolites, and alkaloids derived from ornithine, lysine, and nicotinic acid. The activation of secondary metabolite pathways may reflect plant adaptation to environmental changes, while the regulation of alkaloid biosynthesis influences plant physiology (Kong et al., 2021). Alterations in the carbon metabolism pathway may affect energy metabolism and material circulation within the plant (Wu et al., 2024b). Up-regulated metabolites in T2 were enriched in general metabolic pathways. In T3 treatment, up-regulated metabolites were enriched in tryptophan metabolism and microbial metabolism. Changes in tryptophan metabolism could impact neurotransmitter synthesis and physiological regulation. The microbial metabolism pathways suggest interactions between microbial communities and the host organism (Lueders et al., 2006). These findings indicate that microbial metabolism plays a key role in T3 treatment, with the activation of secondary metabolite pathways reflecting adaptive responses to environmental conditions (Chamkhi et al., 2021).

The 16S and ITS sequencing data further elucidate how planting treatments influence microbial communities (Ge et al., 2023). T1, for example, led to an increase in genes related to transcription, signal transduction, and stress-related processes, which suggests that soil microorganisms were actively responding to the altered rhizosphere environment (Korenblum et al., 2022; Li et al., 2023). Arbuscular mycorrhiza fungi (AMF) is a type of fungus that forms a symbiotic relationship with plant roots, promoting nutrient absorption and plant growth (Santander et al., 2017). The increased abundance of AMF in T1 highlights the role of these beneficial fungi in enhancing nutrient uptake and supporting plant growth through symbiotic relationships (Khan, 2020). This finding is consistent with other studies that emphasize the positive impact of AMF on plant nutrition and stress resilience in agricultural systems (Zhu et al., 2021). Saprophytic fungi, such as undefined saprophytes and wood-decaying fungi, play a key role in decomposing organic matter within ecosystems (Bollmann-Giolai et al., 2022). The increased abundance of these fungi in T1 may indicate more organic matter availability or changes in environmental conditions, fostering their growth (Mishra et al., 2024). The higher abundance of genes related to plant pathogens and endophytes may suggest a shift in the interactions between plants and microorganisms, possibly increasing the pressure from pathogens or altering the plant-endophyte relationship in T1 (Yue et al., 2023).

T2 treatment, which promoted genetic information processing and altered microbial gene replication, indicates an adaptive response to environmental conditions that may favor the proliferation of certain microbial groups (Li et al., 2021). The increased abundance of genes associated with animal pathogens and saprophytes in T2 might point to shifts in ecological niches and microbial competition, which could affect disease dynamics in the soil (Bai et al., 2022). These changes may reflect modifications in ecological niches, making certain fungal groups, such as wood-decaying fungi, more competitive in T2 (Pantigoso et al., 2022). The increased abundance of genes related to animal pathogens, saprophytes, and endophytes may indicate complex interactions between pathogens and fungal communities, or the influence of animal activities on soil microorganisms (Gouda et al., 2018).

T3 treatment revealed changes that favored the growth of dung saprotrophs and animal symbionts, which could be linked to the use of organic fertilizers or changes in soil pH and nutrient availability (Song et al., 2023). In T3, genes related to dung saprotrophs, undefined saprotrophs, and animal endosymbionts were more abundant. The higher abundance of fecal saprophytes and undefined saprotrophs could be linked to organic fertilizer application or increased animal activity under T3 (Qu et al., 2020). This suggests that the microbial community in T3 may have been more adapted to decomposing organic matter, which is a key process in maintaining soil structure and nutrient cycling (Zhou et al., 2023b). The increase in microbial pathways related to metabolism and nutrient acquisition further underscores the importance of microbial interactions in sustaining soil health and promoting plant growth (Yang et al., 2023).

The hierarchical microbial shifts revealed treatment-specific linkages between green manure inputs and functional microbiota assembly. T3’s high-carbon amendment enriched Firmicutes and Fusarium, driving rapid organic matter decomposition, while T1’s *V. myuros* favored acid-tolerant Acidobacteria and Glomeromycota, enhancing symbiotic nutrient acquisition. T2 uniquely selected Chloroflexi and Entrophospora, targeting recalcitrant lignin degradation. Bacterial communities exhibited stronger treatment divergence than fungi, where Ascomycota persistently dominated, suggesting bacteria better reflect short-term organic input changes. These contrasts highlight trade-offs: T3 prioritizes decomposition, T1/T2 enhance symbiosis, and crop-specific taxa selection guides precision soil management.

Implications for Sustainable Agriculture: The findings of this study align with the broader literature on the importance of crop rotation and intercropping for soil health (Kong et al., 2021). Intercropping with *V. myuros* and rotating with crops like rape offer clear benefits in terms of improving soil structure, reducing pest and disease incidence, and enhancing nutrient cycling (Yang et al., 2024). However, the excessive reliance on compound fertilizers, as highlighted in the study, poses risks of nutrient accumulation and soil degradation, which can impair long-term soil fertility (Liu et al., 2020). This underscores the need for balanced fertilizer use and careful management of organic and chemical inputs in tobacco farming to optimize both economic returns and environmental sustainability (Feng et al., 2023).

## 5 Conclusion

This study systematically investigated the effects of different tobacco cropping systems on soil rhizosphere ecology through integrated analysis of microbial communities and metabolite profiles. The results demonstrated that intercropping with *V. myuros* (T1) significantly enhanced symbiotic fungal associations (particularly Glomeromycota), while rotation with rape (T2) promoted the enrichment of lignin-degrading microbial consortia (Basidiomycota and Chloroflexi). The application of fermented cake fertilizer (T3) substantially increased microbial diversity and fostered cooperative metabolic networks, particularly in organic matter decomposition pathways. Metabolomic analysis revealed treatment-specific regulation of key metabolic pathways including phenylpropanoid biosynthesis, carbon metabolism, and amino acid metabolism, highlighting their crucial roles in nutrient cycling and plant-microbe interactions. These findings provide strong evidence that diversified cropping systems offer superior benefits over continuous monoculture, including improved soil health, enhanced nutrient availability, and better disease resistance while reducing dependence on synthetic fertilizers. The study suggests that optimal soil management strategies should be tailored to specific agricultural contexts, combining organic amendments with microbial conservation practices. Future research should focus on long-term field trials to validate these findings and develop practical guidelines for sustainable tobacco cultivation. This work contributes to our understanding of rhizosphere ecology and provides a scientific basis for developing more sustainable agricultural practices.

## Data Availability

The data that support the findings of this study are available on request from the corresponding author upon reasonable request. 16S-seq and ITS-seq raw data have been deposited in the NCBI sequence read archive (SRA) under project accession PRJNA1252381 and PRJNA1252388. The raw data for the metabolome of this study have been uploaded to MetaboLights (Number: MTBLS10471).
