# Supplementary material for: Comprehensive metabolomic and microbial analysis of tobacco rhizosphere soil responses to crop rotation and fertilization
Source: Front Plant Sci. 2025 Jun 3;16:1595870. doi: 10.3389/fpls.2025.1595870 (PMC12170533; doi:10.3389/fpls.2025.1595870)
Supplement: Supplementary file 1 [file DataSheet1.pdf]

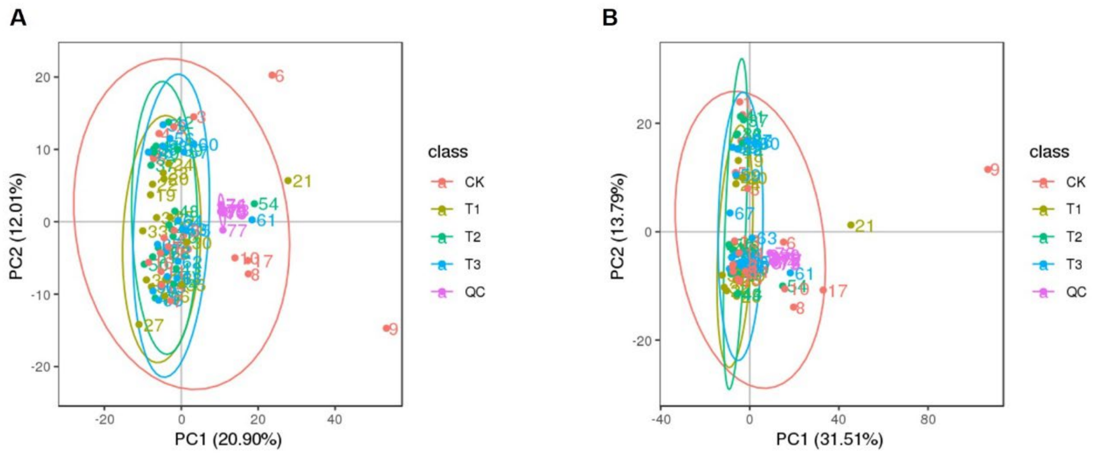

Figure S1. PCA analysis of experimental samples and QC samples. The composition of negative(A) and positive(B) rhizosphere metabolites. The abscissa, PC1, and the ordinate, PC2, respectively represent the scores of the first - and second - principal components in ranking. The scattered points of different colors represent samples of different experimental groups (CK: Treatment control, T1: Rotation + Intercropping group, T2: Rotation group, T3: Cake fertilizer group). The ellipse is the 95% confidence interval. A more - aggregated QC indicates better stability of the detection process, reflecting the consistency and reliability of the analytical method. This graphical representation provides a visual assessment of the variability and similarity among the experimental groups and the quality control samples.

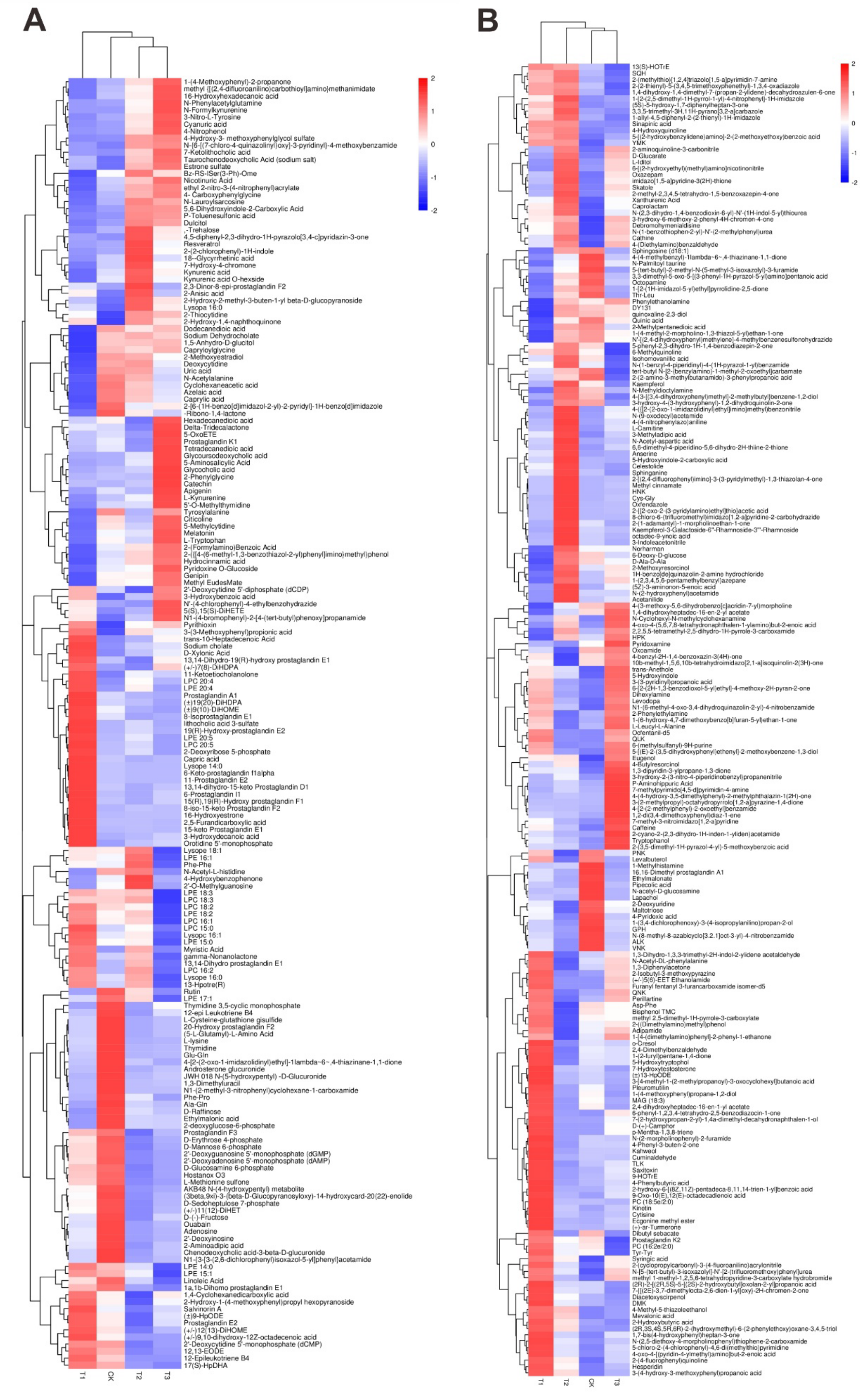

Figure S2. Cluster heat map of total differential metabolites (A) negative metabolites

heatmap. (B) positive metabolites heatmap. Perform hierarchical cluster analysis on the differential metabolites between each comparison pair, normalize and cluster the relative quantitative values of differential metabolites. The horizontal direction is the clustering of metabolites, and the vertical direction is the sample grouping. The shorter the clustering branch, the higher the similarity.

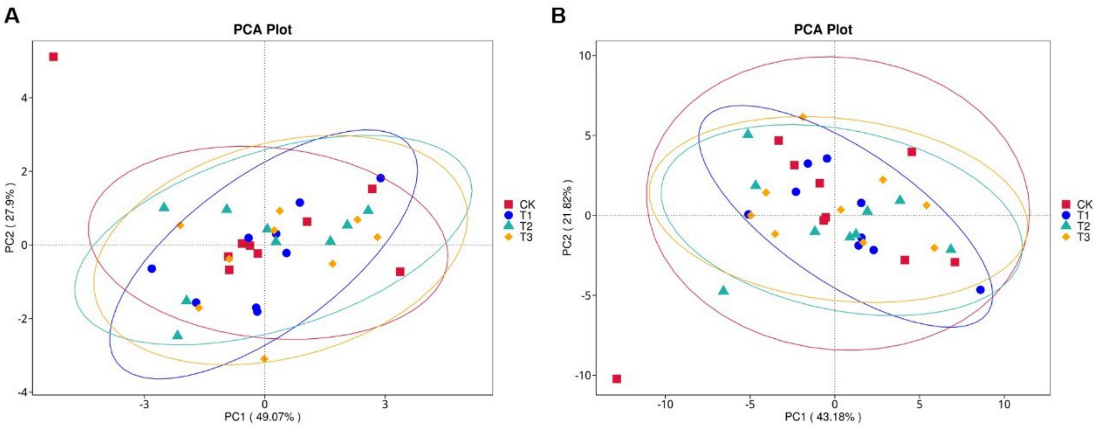

Figure S3. The results of PCA analysis for each function in 16S sequencing, displaying the same beta PCA. Based on the abundance statistics of functional annotations from the database, PCA (Principal Component Analysis) dimensionality reduction analysis is performed. (A) The PCA analysis results of the Level1 database. (B) The PCA analysis results of the Level2 database. If the functional composition of the samples is more similar, their distances in the reduced-dimensional plot will be closer. The horizontal coordinate represents the first principal component, and the percentage indicates the contribution of the first principal component to the sample differences; the vertical coordinate represents the second principal component, and the percentage shows the contribution of the second principal component to the sample differences; each point in the plot represents a sample, and samples from the same group are indicated in the same color. CK: Treatment control, T1: Rotation + Intercropping group, T2: Rotation group, T3: Cake fertilizer group.

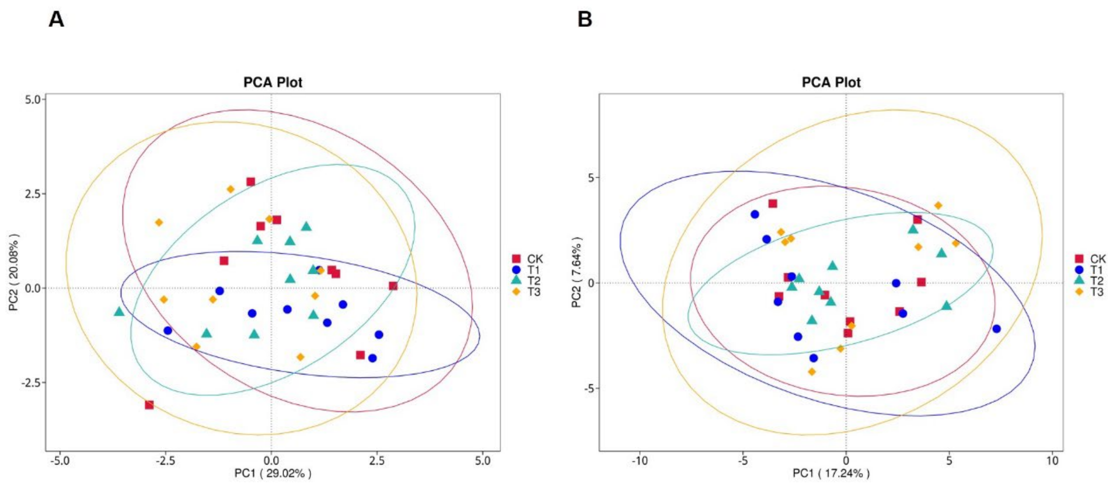

Figure S4. The results of PCA analysis for each function in ITS sequencing, displaying the same beta PCA. Based on the abundance statistical results of functional annotations from the database, PCA (Principal Component Analysis) dimensionality reduction analysis is performed. (A) The PCA analysis results of the mode database. (B) The PCA analysis results of the guild database. If the functional composition of the samples is more similar, their distances in the reduced-dimensional plot will be closer. The horizontal coordinate represents the first principal component, and the percentage indicates the contribution of the first principal component to the sample differences; the vertical coordinate represents the second principal component, and the percentage indicates the contribution of the second principal component to the sample differences; each point in the plot represents a sample, and samples from the same group are colored the same. CK: Treatment control, T1: Rotation + Intercropping group, T2: Rotation group, T3: Cake fertilizer group.

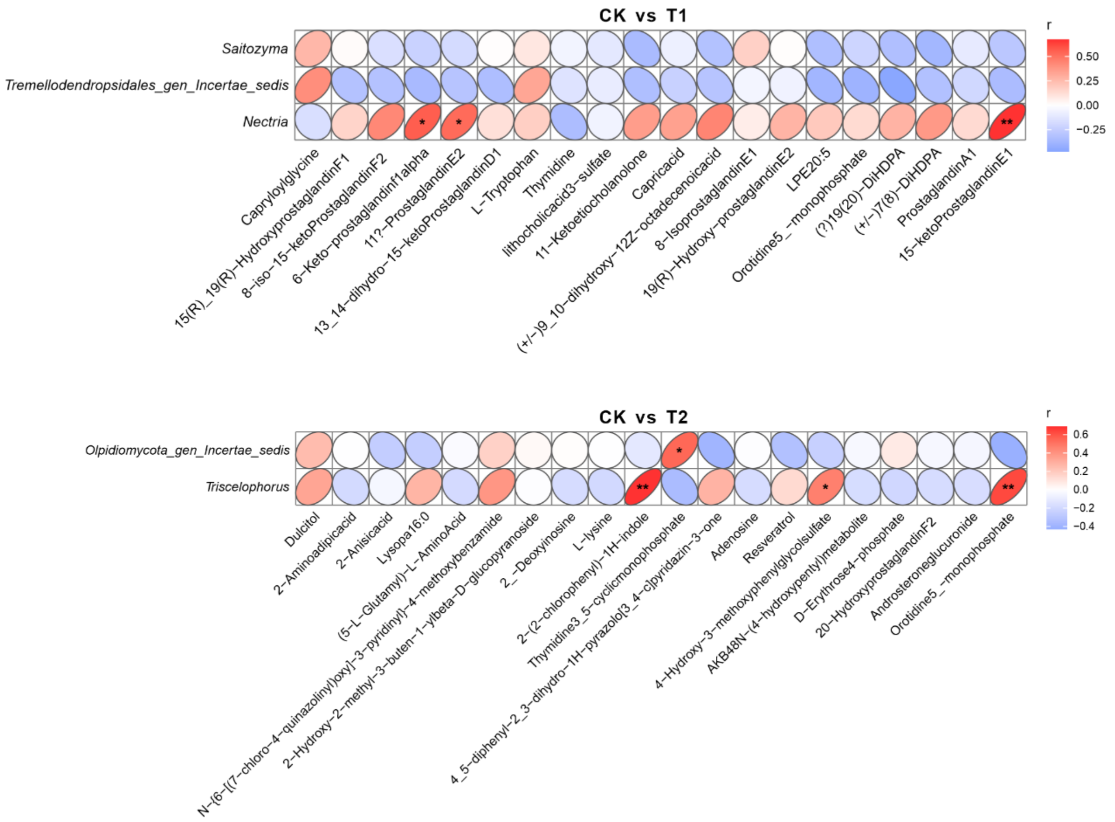

Figure S5. Spearman correlation heatmap of ITS-based fungal taxa and top VIP-selected metabolites across fertilization regimes. Rows represent fungal genera; columns display metabolites. Red/blue ellipses denote positive/negative correlations ($|r| \geq 0.25$, $p < 0.05$), with ellipse thinness inversely proportional to $|r|$ magnitude. Blank cells indicate non-significant associations ($p \geq 0.05$).

Table S1. The primer sequences used for each region.

Type	Region	Primer Sequences
Bacteria	16Sv4	GTGCCAGCMGCCGCGGTAA, GGACTACHVGGGTWTCTAAT
Bacteria	16Sv34	CCTAYGGGRBGCASCAG, GGACTACNNGGGTATCTAAT
Bacteria	16Sv45	GTGCCAGCMGCCGCGGTAA, CCGTCAATTCCTTTGAGTTT
Bacteria	16Sv57	AACMGGATTAGATACCCKG, ACGTCATCCCCACCTTCC
Eukaryotes	18Sv4	GCGGTAATTCCAGCTCCAA, AATCCRAGAATTTCACCTCT
Fungi	ITS1-1F	CTTGGTCATTTAGAGGAAGTAA, GCTGCGTTCTTCATCGATGC
Fungi	ITS1-5F	GGAAGTAAAAGTCGTAACAAGG, GCTGCGTTCTTCATCGATGC
Fungi	ITS2	GCATCGATGAAGAACGCAGC, TCCTCCGCTTATTGATATGC
Novel Archaea	Novel-Archaea-V4	CAGCCGCCGCGGTAA, GTGCTCCCCCGCCAATTCCT

Table S2. Anosim intergroup difference analysis

Group	R-value	P-value
CK-T1	-0.04938	0.709
CK-T2	-0.0727	0.861
CK-T3	-0.11488	0.993
T1-T2	0.01526	0.288
T1-T3	0.00892	0.313
T2-T3	-0.05573	0.735

Table S3. Adonis intergroup difference analysis

Group	Df	SumsOfSqs	MeanSqs	F.Model	R2	Pr(>F)
CK-T1	1(16)	0.13228(2.4509)	0.13228(0.15318)	0.86354	0.05121(0.94879)	0.554
CK-T2	1(16)	0.12421(2.63074)	0.12421(0.16442)	0.75544	0.04509(0.95491)	0.762
CK-T3	1(16)	0.09243(2.6106)	0.09243(0.16316)	0.56651	0.0342(0.9658)	0.975
T1-T2	1(16)	0.15418(2.47411)	0.15418(0.15463)	0.99709	0.05866(0.94134)	0.341
T1-T3	1(16)	0.15511(2.45397)	0.15511(0.15337)	1.0113	0.05945(0.94055)	0.335
T2-T3	1(16)	0.12361(2.63381)	0.12361(0.16461)	0.7509	0.04483(0.95517)	0.75

Table S4. Anosim intergroup difference analysis

Group	R-value	P-value
CK-T1	0.0439	0.222
CK-T2	0.0715	0.147
CK-T3	-0.06481	0.844
T1-T2	0.00206	0.386
T1-T3	0.04938	0.21
T2-T3	0.04184	0.23

Table S5. Adonis intergroup difference analysis

Group	Df	SumsOfSqs	MeanSqs	F.Model	R2	Pr(>F)
CK-T1	1(16)	0.1618(2.37645)	0.1618(0.14853)	1.08937	0.06375(0.93625)	0.315
CK-T2	1(16)	0.16408(2.22813)	0.16408(0.13926)	1.17821	0.06859(0.93141)	0.25
CK-T3	1(16)	0.09187(2.48418)	0.09187(0.15526)	0.59174	0.03566(0.96434)	0.94
T1-T2	1(16)	0.12285(1.94339)	0.12285(0.12146)	1.01144	0.05946(0.94054)	0.391
T1-T3	1(16)	0.17828(2.19944)	0.17828(0.13747)	1.29694	0.07498(0.92502)	0.199
T2-T3	1(16)	0.15163(2.05112)	0.15163(0.1282)	1.18278	0.06884(0.93116)	0.256
